# Nurses’ Clinical Alarm–Related Behaviors and Their Associated Factors in Malawian Critical Care Units: A Cross‐Sectional Study

**DOI:** 10.1155/jonm/9349880

**Published:** 2026-09-22

**Authors:** Chimwemwe Joyce Kamuyalo, Bingyu Li, Elihuruma Eliufoo Stephano, Mtoro J. Mtoro, Liqing Yue

**Affiliations:** ^1^ Teaching and Research Section of Clinical Nursing, Xiangya Hospital of Central South University, Changsha, Hunan, China, csu.edu.cn; ^2^ Department of Paediatrics, Mchinji District Hospital, Ministry of Health, Lilongwe, Malawi, sante.gov.ma; ^3^ Xiangya School of Nursing, Central South University, Changsha, Hunan, China, csu.edu.cn; ^4^ Clinical Nursing Teaching and Research Section, The Second Xiangya Hospital, Central South University, Changsha Hunan, 410011, China, csu.edu.cn; ^5^ TILAM International, Dar es Salaam, Tanzania

**Keywords:** behaviors, clinical alarms, critical care units, Malawian, nurses

## Abstract

**Background:**

Understanding nurses’ clinical alarm–related behaviors is crucial for enhancing patient safety in critical care settings. These settings operate under significant constraints that profoundly influence how nurses prioritize and respond to alarms. While suboptimal alarm management has been extensively studied in well‐resourced settings, research remains scarce in resource‐constrained settings. This study aimed to evaluate nurses’ clinical alarm–related behaviors and their associated factors in critical care units within resource‐limited contexts.

**Methods:**

An analytical cross‐sectional study was conducted among 434 nurses recruited via multistage stratified random sampling from five hospitals in Malawi. Data were collected using validated scales for alarm behaviors, knowledge, attitudes, fatigue, burnout, and resilience measurements. Data were analyzed using descriptive statistics, Pearson’s correlation, and multiple linear regression with backward elimination to identify independent factors influencing clinical alarm–related behavioral scores, with statistical significance set at *p* < 0.05.

**Results:**

The mean clinical alarm–related behavioral score was 68.89 ± 9.72 out of 85. Factors independently associated with higher behavioral scores included working in the ICU (*β* = 5.47, 95% CI: 0.61 to 10.33, *p* *= *0.027), resilience (*β* = 0.18, 95% CI: 0.07 to 0.29, *p* = 0.001), positive attitude (*β* = 0.36, 95% CI: 0.21 to 0.51, *p* = 0.001), implemented alarm management protocol (*β* = 4.54, 95% CI: 2.83 to 6.24, *p* < 0.001), and having experienced alarm–related adverse events in the last 2 years (*β* = 2.02, 95% CI: 0.28 to 3.75, *p* = 0.023). Conversely, greater years of nursing experience (*β* = −0.39, 95% CI: −0.75 to −0.03, *p* = 0.032) and higher knowledge scores (*β* = −0.93, 95% CI: −1.47 to −0.38, *p* < 0.001) were associated with lower behavioral scores. The model explained a substantial proportion of the variance in alarm‐related behaviors (*R* ^2^ = 0.673).

**Conclusion:**

These results indicate that effective alarm‐related behaviors extend beyond technical knowledge and competence and require multifaceted support. Interventions may benefit from comprehensive, system‐level strategies that integrate standardized protocols, tailored training to bridge knowledge‐practice gaps, resilience‐building program, and efforts to foster positive attitudinal shifts among nurses. Such targeted approaches hold strong potential to optimize clinical alarm–related behaviors, mitigate risks such as delayed responses, and ultimately enhance patient safety in resource‐limited critical care environments.

**Implications for Nursing Management:**

The findings of this study call for nurse managers and hospital administrators to consider implementing debriefing protocols following any alarm‐related incident. By reinforcing positive attitudes (through leadership, peer support, and recognition) and integrating resilience‐building programs, these strategies may enhance proactive alarm behaviors and ultimately improve patient safety in resource‐limited critical care settings.

**Summary**
• What does this study contribute to the wider global clinical community?
◦ This study offers the first quantitative evidence of nurses’ self‐reported alarm‐related behaviors and their associated factors in Malawian adult critical care, a setting not previously characterized in the literature dominated by well‐resourced contexts. Behavioral performance was moderate overall and broadly comparable to levels documented in high‐income countries, yet shaped by distinct resource constraints.
◦ Contrary to common expectations, more years of experience and higher knowledge levels were each independently associated with lower clinical alarm–related behavioral scores, suggesting possible desensitization among experienced nurses and a knowledge‐practice gap. In contrast, resilience and positive attitudes were positively associated with higher behavioral scores, indicating that psychological factors influence alarm management performance beyond technical competence.
◦ These findings support system‐level strategies, including standardized alarm management protocols, attitude‐focused education, resilience‐building programs, and specialized refresher training for experienced nurses. Specifically, it is tailored to the resource constraints of critical care settings in low‐ and middle‐income countries, with potential relevance for improving patient safety in similar contexts globally.

## 1. Introduction

Clinical alarms are safety‐critical features of physiologic monitoring in critical care units, designed to draw clinicians’ attention to clinically meaningful changes in a patient’s status, thereby mitigating adverse events [1]. However, in everyday practice, alarms occur frequently and in high volumes, and a substantial proportion are false or nonactionable (commonly reported in the range of 70%–90%) [2]. This high alarm burden precipitates alarm fatigue, whereby repeated exposure to low‐value alerts may desensitize staff, erode trust in alarm systems, and delay recognition of and response to clinically important alarms [3]. Given these implications for patient safety, alarm fatigue has been identified repeatedly as a major health technology hazard [2, 4]. Studies across different countries have reported moderate to high levels of alarm fatigue among critical care nurses, compromising their vigilance, prioritization, and timely responses to alarms [5–8]. These effects ultimately manifest in routine alarm management practices, making nurses’ clinical alarm–related behavior a critical target for assessment and improvement.

In sub‐Saharan Africa (SSA), critical care is delivered under formidable constraints, including limited resources, staffing shortages, and an evolving healthcare infrastructure [9]. Simultaneously, the adoption of monitoring technologies capable of generating clinical alarms is accelerating across the region [10]. In settings where standardized protocols, structured training, and technical support are limited, greater alarm exposure disproportionately increases nursing workload and jeopardizes safe alarm handling. Mwale et al. (2025) examined the barriers and facilitators to implementing a continuous monitoring system in pediatric critical care in Malawi, and broader SSA reviews have documented structural challenges in critical care delivery [11]. However, to our knowledge, no study has quantitatively characterized nurses’ alarm‐related behaviors or their associated factors in Malawian critical care units. Farajalla et al. similarly documented the scarcity of alarm management research in low‐ and middle‐income country (LMIC) settings, underscoring the need for context specific evidence [12]. These contextual features suggest that alarm management challenges and feasible solutions in Malawi may diverge significantly from those described in high‐income settings, warranting locally generated, practice‐focused evidence to guide improvements in Malawian critical‐care units [13].

Globally, research has identified multiple contributors to suboptimal clinical alarm–related behaviors, including high alarm volume, inappropriate parameter customization, lack of standardized alarm management protocols, workflow interruptions, and insufficient staffing [5, 14–16]. Accordingly, interventions have typically combined governance and policy measures (for example, standardized protocols), targeted training, and technological optimization to reduce low‐value alarms and improve alarm reliability [16, 17]. However, much of the evidence originates from high‐income settings, where staffing models, training infrastructure, device maintenance, and governance mechanisms may differ substantially from those in resource‐limited environments, such as Malawi. Consequently, the transferability of known risk factors and intervention approaches to Malawian critical care units should be evaluated rather than assumed to be applicable. In addition, alarm management occurs in demanding work environments [14]. Sustained workload and recurrent interruptions may contribute to burnout, whereas psychological resources, such as resilience, may buffer these effects and support vigilant and timely alarm responses, thereby influencing alarm‐related behaviors.

Despite widespread recognition of alarm management and related safety concerns, analytical evidence remains limited regarding which individual and organizational factors predict nurses’ routine alarm‐related behaviors in Malawian critical care settings. In this study, clinical alarm–related behaviors refer to nurses’ self‐reported routine practices for managing clinical alarms in everyday critical care work following the framework proposed by Liao et al. [18]. These behaviors encompass five key domains: (1) alarm settings (customizing parameters to patient condition), (2) alarm notifications (detecting alarm signals), (3) alarm recognition (identifying source and priority), (4) alarm responses (acknowledgment, prioritization, troubleshooting, escalation, handling false alarms), and (5) alarm learning (seeking knowledge and reflecting on alarm events).

From a human factors and systems perspective, the Systems Engineering Initiative for Patient Safety (SEIPS) model conceptualizes clinical alarm–related behaviors as emerging from the interaction between person‐level factors, cognitive resources (knowledge), attitudinal dispositions, and psychological capital (resilience and burnout), and organizational and environmental conditions such as protocol availability, departmental alarm burden, and training support [19]. Guided by this framework, this study aimed to describe nurses’ clinical alarm–related behaviors in Malawian critical care units and identify factors associated with these behaviors, including sociodemographic characteristics, organizational factors, alarm‐related knowledge and attitudes, alarm fatigue, burnout, and resilience. Clarifying actionable factors within this local context may inform the development or strengthening of standardized alarm management protocols, targeted training, and supportive strategies to enhance patient safety and improve nurses’ working conditions in Malawi and similar SSA settings [20].

## 2. Materials and Methods

### 2.1. Study Design

An analytical cross‐sectional study design was employed to investigate nurses’ clinical alarm–related behaviors and to identify their associated factors. A cross‐sectional design was selected as the most appropriate approach for this population, given the complete absence of prior baseline data on clinical alarm–related behaviors in Malawian critical care. Cross‐sectional designs are widely used and recommended for prevalence estimation and hypothesis generation in understudied clinical contexts.

### 2.2. Study Site and Settings

Data were collected from September 2025 to November 2025 from five hospitals representing Malawi’s major administrative zones: four public tertiary referral hospitals and one private hospital. Public hospitals serve as major referral centers and academic institutions, while private hospitals were included to ensure variation in institutional characteristics, staffing patterns, and resource availability. All participating hospitals provided critical care services and were equipped with patient monitoring systems capable of generating clinical alarms.

### 2.3. Study Participants

This study focused on nurses, who are the largest segment of frontline healthcare providers in critical care units. Participants were recruited from various critical care units, including the neonatal intensive care unit (NICU), pediatric intensive care unit (PICU), postanesthesia care unit (PACU), and general intensive care unit (ICU). These units were chosen because of their high‐acuity environment, where patients require continuous and intensive monitoring.

The inclusion criteria required nurses to be actively working in the selected units with at least 1 year of experience in critical care and willing to provide informed consent. The exclusion criteria applied to nurses who declined participation or were currently on any type of leave (study, annual, sick, or maternity) during the data collection period.

### 2.4. Sample Size

The sample size was determined using the Cochran’s formula [7, 21]. Given the absence of prior studies on alarm‐related behaviors in Malawi, a conservative proportion of 50% was assumed in this study. With a 5% margin of error and 95% confidence interval, the initial requirement was 384 participants. To account for potential nonresponse, an additional 10% was added, yielding a target of 422 participants. Ultimately, 434 nurses were recruited.

### 2.5. Sampling Procedure

A multistage stratified sampling technique was used to ensure a representative sample. Initially, hospitals were stratified into public and private hospitals to account for operational differences in staffing and resources. The overall sample was allocated proportionally (approximately 70% public and 30% private schools). Within each hospital, each critical care unit was treated as a separate stratum to ensure comprehensive representation of the sample. The number of nurses selected from each unit was determined proportionally to the unit’s staffing size. Nurse lists were obtained from the human resources offices of each participating hospital before data collection. These lists included all nurses currently assigned to critical care units. Simple random sampling was applied using a random number generator. The overall response rate was 96.4% (434 of 450 approached).

The final sample comprised 369 nurses from public hospitals (85.0%) and 65 from private hospitals (15.0%). This deviation from the initial proportional allocation occurred because the private hospital had a smaller eligible nurse population than anticipated during planning, and site‐specific response rates (while high overall at 96.4%) resulted in fewer private‐sector participants despite proportional targeting and adjustments for nonresponse within the hospitals. No replacement sampling from outside the selected facilities was performed to preserve the multistage stratified design and representativeness of the included institutions.

### 2.6. Study Variables

#### 2.6.1. Dependent Variable

Nurses’ clinical alarm–related behaviors were measured as a composite continuous variable using a 17‐item behavior scale comprising five dimensions: alarm settings (five items), alarm notifications (two items), alarm recognition (two items), alarm responses (seven items), and alarm learning (one item) [18]. Items were rated on a five‐point Likert scale (1 = “*never*,” and 5 = “*always*”). Negatively worded items were reverse scored prior to summation, so that higher total scores indicated better self‐reported alarm‐related behaviors. Total scores were computed by summing all 17 items (range: 17–85). Domain‐specific scores were also calculated to enable a dimension‐level examination of performance.

#### 2.6.2. Independent Variables

The independent variables included sociodemographic and professional characteristics (age group, sex, education level, and professional title), work context (unit type and sector), experience (overall nursing experience category and years of critical care experience as a continuous variable), nurse–patient ratio (patients assigned per shift), and alarm management context (prior formal alarm training, availability of alarm management protocols in the department within the past 2 years, and alarm‐related adverse events experienced in the past 2 years; all coded yes/no). Psychological and cognitive factors (knowledge, attitude, alarm fatigue, burnout, and resilience) were included as independent variables based on the scale.

### 2.7. Measurement Tools

A structured questionnaire with five sections was employed, beginning with demographic and professional characteristics of the respondents. The subsequent sections utilize validated scales to comprehensively measure alarm‐related constructs.

Nurses’ clinical alarm–related behaviors were assessed using a 17‐item scale developed by Liao et al. [18]. The scale covers five dimensions: alarm settings, notifications, recognition, responses, and learning. These domains were integrated into a composite score (range, 17–85). In the present study, the scale demonstrated an acceptable internal consistency (Cronbach’s *α* = 0.80). Negatively worded items were reverse scored (1 = always becomes 5 and 5 = never becomes 1) prior to summation, so that higher scores consistently reflected better alarm‐related behaviors across all 17 items. Domain‐level scores are presented in Table 1, while the composite score was used for the regression analyses.

**TABLE 1:** Scores of the domains of nurses’ clinical alarm–related behaviors (*n* = 434).

Behavioral items	Mean (±SD)
Dimension 1: Alarm settings	19.40 ± 4.74
1. During shift handovers, I check patients′ alarm settings on devices.	4.10 ± 1.19
2. I adjust alarm volume based on the unit environment and time of day.	3.56 ± 1.44
3. When using alarm‐enabled devices, I adjust alarm settings.	4.00 ± 1.08
4. When a patient’s condition changes, I adjust alarm settings.	3.75 ± 1.28
5. I set personalized alarm thresholds based on patient conditions.	3.97 ± 1.15
Mean score in Dimension 1 per item	3.88 ± 1.23
Dimension 2: Alarm notifications	5.96 ± 2.43
1. I become aware of alarms through patient or caregiver reminders.	3.15 ± 1.42
^∗^ 2. I have missed alarms due to not hearing the alarm sound.	2.81 ± 1.40
Mean score in Dimension 2 per item	2.98 ± 1.41
Dimension 3: Alarm recognition	8.24 ± 1.98
1. I can accurately identify the source device of an alarm based on its signal.	4.04 ± 1.13
2. Based on alarm signals, I can distinguish between different alarm priority levels.	4.20 ± 1.08
Mean score in Dimension 3 per item	4.12 ± 1.11
Dimension 4: Alarm responses	31.12 ± 4.62
1. I respond to all alarms in my assigned area.	4.34 ± 0.97
2. Upon receiving an alarm signal, I respond immediately.	4.46 ± 0.88
3. When multiple alarms occur simultaneously, I prioritize responses based on alarm levels.	4.38 ± 0.97
4. I can correctly handle the events triggering alarms.	4.29 ± 0.95
^∗^ 5. If I believe an alarm is false or a nuisance alarm, I do not respond.	2.77 ± 1.44
^∗^ 6. When false or nuisance alarms occur frequently, I mute or turn off alarms.	2.79 ± 1.46
7. For alarms I cannot handle, I actively seek help from colleagues.	4.27 ± 1.12
Mean score in Dimension 4 per item	3.90 ± 1.14
Dimension 5: Alarm learning	4.17 ± 1.11
1. I actively seek ways to acquire alarm‐related knowledge.	4.17 ± 1.11
Mean score in Dimension 5 per item	4.17 ± 1.11
Total behavior mean score per item	3.83 ± 1.20
Total sum behavior score	68.89 ± 9.72

Abbreviation: SD, standard deviation.

^∗^Reverse scored.

Alarm knowledge was measured using 12 multiple‐choice questions, with one point awarded for each correct answer [18]. In the current study, the knowledge scale showed acceptable reliability with a Kuder–Richardson 20 (KR‐20) of 0.78.

Alarm attitude was assessed using an 11‐item, 5‐point Likert scale, with higher scores indicating more positive attitudes towards clinical alarms [7]. The total score is calculated by summing the scores of all items and ranges from 0 to 124, with a higher score indicating a higher level of alarm fatigue.

Burnout was quantified using the Maslach Burnout Inventory (MBI) across three dimensions: emotional exhaustion, depersonalization, and personal accomplishment, with a total score up to 132 [7].

Resilience was measured using the 10‐item Connor‐Davidson Resilience Scale (CD‐RISC‐10) [22], with scores ranging from 0 to 40, with higher scores indicating greater resilience. In the current study, all instruments demonstrated acceptable psychometric properties, with Cronbach’s alpha coefficients ranging from 0.77 to 0.96 for internal consistency and test–retest reliability exceeding 0.81, indicating good reliability.

Existing questionnaires, particularly those on alarm fatigue, burnout, and resilience, were adapted to ensure contextual and cultural appropriateness for the Malawian clinical environment. Adjustments included language and terminology modifications to reflect local expressions, contextual refinements for realistic ICU scenarios, and the incorporation of additional relevant sociodemographic variables, such as the nurse‐patient ratio.

The knowledge, attitude, and behavior questionnaire on nurses’ clinical alarm–related behaviors used in this study was originally developed in Chinese. The questionnaire was authorized by the original developer prior to use. It was translated into English, culturally adapted, and tested for reliability following the WHO translation guidelines [23]. The translation involved forward and backward translations by two bilingual nursing experts. Minor modifications were made to enhance cultural relevance and clarity, and content validity was evaluated by an expert panel (Content Validity Index = 0.92). Prior to final use, the prefinal version was pilot tested with 39 intensive care nurses, who were not included in the final analysis. Finally, the adapted questionnaire demonstrated good reliability, with KR‐20 = 0.78 for knowledge and Cronbach’s alpha of 0.88 and 0.80 for attitude and nurses’ alarm‐related behavior, respectively.

### 2.8. Data Collection Procedure

Data were collected from September to November 2025 using a secure Kobo Collect electronic questionnaire by four trained research assistants. Eligibility was confirmed, and electronic informed consent was obtained prior to their participation. The participants completed the questionnaire at their convenience. Survey links were shared via WhatsApp with randomly selected nurses. Completed responses were uploaded to a password‐protected server that was accessible only to the research team.

To mitigate social desirability bias, participants were assured of full anonymity and confidentiality; responses were collected electronically without direct researcher observation, and no identifying information was linked to the individual responses. Recall bias was minimized by anchoring questionnaire items to nurses’ current routine practice rather than past specific events. Despite these precautions, the possibility of over‐reporting socially desirable behaviors cannot be entirely excluded, and findings should be interpreted with this limitation in mind.

### 2.9. Data Analysis

The data were cleaned and analyzed using STATA Version 18. Categorical variables are presented as frequencies and percentages, and continuous variables are summarized as means and standard deviations. The normality of continuous variables was assessed using the Kolmogorov–Smirnov test, and parametric methods were used where the assumptions were met.

Bivariate analyses compared mean behavior scores using independent‐samples *t*‐tests (binary factors) and one‐way analysis of variance (ANOVA) (factors with > 2 categories). Pearson’s correlation was used to assess the linear associations between the behavior scores and continuous factors. Variables with *p* < 0.25 in the bivariate analyses were entered into a multivariable linear regression model; age was retained in the model regardless of bivariate significance as a potential confounder.

Variable selection was guided primarily by the SEIPS framework, ensuring the inclusion of relevant person‐level, organizational, and environmental factors. All theoretically relevant variables were initially entered into a multiple linear regression model. Backward elimination was then applied as a secondary step to derive a parsimonious final model while retaining the important confounders. The regression assumptions were thoroughly evaluated and adequately met. Linearity was confirmed by examining scatterplots of residuals versus predicted values, which showed no systematic patterns. The normality of the residuals was assessed using the Shapiro–Wilk test (*W* = 0.987, *p* = 0.062) and visual inspection of Q‐Q plots, both of which supported approximate normality. Homoscedasticity was verified using the Breusch–Pagan test (*χ* ^2^ = 8.76, df = 12, *p* = 0.721) and residual‐versus‐fitted plots, which indicated constant variance. No influential observations were detected, and all Cook’s distance values were below 1 (maximum Cook’s distance = 0.042). Multicollinearity among independent variables was assessed using variance inflation factors (VIFs) prior to entering the factors into the multivariate linear regression model. Alarm fatigue was excluded from the final multivariable model because of its exceptionally high intercorrelation with other predictors (particularly burnout, *r* = 0.6095) and the resulting multicollinearity (VIF = 12.8 when forced into the model). All other retained factors in the final model demonstrated acceptable VIF values (< 3.0), indicating no problematic multicollinearity (resilience VIF = 1.45, attitude VIF = 1.32, knowledge VIF = 1.68, years of nursing experience VIF = 2.1, and implemented protocols VIF = 1.9).

Missing data were minimal (< 1% of the items across all variables). Cases with any missing variables were excluded from the regression analyses using complete‐case analysis; no imputation was performed given the negligible extent of missingness. Statistical significance was set at *p* < 0.05 for all analyses.

## 3. Results

### 3.1. Associations Between Sociodemographic Characteristics and Nurses Clinical Alarm–Related Behaviors

As summarized in Table 2, among the 434 nurses, more than half were female (59.4%), and the largest age group was 25–34 years old (63.8%). Most participants held a bachelor’s degree (64.1%) and were junior nurses (51.6%). Over half of the respondents were stationed in the ICU (52.7%), and 85% were employed in the public sector. The mean nursing experience was 5.02 ± 3.93 years, and the mean critical care experience was 2.81 ± 2.22 years. Regarding institutional support, 40.1% of the respondents had received formal alarm management training, and 50.9% reported the presence of alarm management protocols. Notably, 62.9% of respondents indicated that their institution had experienced alarm‐related adverse patient events in the past 2 years.

**TABLE 2:** Associations between sociodemographic characteristics and nurses’ clinical alarm–related behaviors (*N* = 434).

Variable	*n* (%)	Total behavior score		*p* value
		Score (mean ± SD)	Test statistics	
Gender			*t* = 0.6564	0.511
Female	258 (59.4)	69.14 ± 8.72		
Male	176 (40.6)	68.51 ± 11.04		
Age			*F* = 0.85	0.430
19–24	93 (21.4)	70.04 ± 8.03		
25–34	277 (63.8)	68.59 ± 9.81		
≥ 35	64 (14.7)	68.47 ± 11.47		
Education			*F* = 1.35	0.261
Diploma	136 (31.3)	68.83 ± 9.41		
Bachelor	278 (64.1)	69.46 ± 9.91		
Masters	20 (4.6)	65.65 ± 12.39		
Professional title			*F* = 3.38	0.018
Enrolled nurse	173 (39.9)	69.66 ± 9.82		
Junior nurse	224 (51.6)	68.48 ± 9.20		
Senior nurse	27 (6.2)	70.41 ± 10.93		
Charge nurse	10 (2.3)	60.30 ± 12.55		
Department			*F* = 8.38	< 0.001
PACU	143 (33.0)	66.69 ± 9.12		
ICU	229 (52.7)	70.95 ± 9.52		
NICU	49 (11.3)	67.16 ± 8.99		
PICU	13 (3.0)	63.00 ± 13.84		
Sector			*t* = 1.12	0.265
Public	369 (85.0)	68.66 ± 9.96		
Private	65 (15.0)	70.12 ± 8.19		
Nursing experience (years)	5.02 ± 3.93			
Years working in the critical care unit	2.81 ± 2.22			
Patients are assigned on a typical shift	4.79 ± 6.72			
Received formal training on alarm management			*t* = −4.12	0.001
Yes	174 (40.1)	71.19 ± 8.07		
No	260 (59.9)	67.33 ± 10.42		
Implemented alarm management protocols			*t* = −7.215	0.001
No	213 (49.1)	63.4 ± 9.8		
Yes	221 (50.9)	71.2 ± 8.1		
Experienced adverse events in last 2 years related to clinical alarms			*t* = −4.0142	< 0.001
No	161 (37.1)	66.48 ± 9.43		
Yes	273 (63.0)	70.30 ± 9.63		

Bivariate analysis revealed that the department of work was significantly associated with clinical alarm–related behavioral scores (*F* = 8.38, *p* < 0.001), with ICU nurses demonstrating the highest total behavioral scores (70.95 ± 9.52). Higher behavioral scores were also significantly associated with having received formal alarm management training (*t* = −4.12, *p* < 0.001) and working in departments with implemented alarm management protocols (*t* = −7.22, *p* < 0.001) and a history of institutional alarm‐related adverse events (*t* = −4.01, *p* < 0.001) (Table 2).

### 3.2. Scores of Clinical Alarm–Related Behaviors

Table 1 presents the scores for clinical alarm–related behaviors. The total behavioral score was 68.89 ± 9.72 (out of 85). When analyzed by dimension, the highest mean scores per item were observed for alarm learning (4.17 ± 1.11) and alarm recognition (4.12 ± 1.11), whereas alarm notifications (2.98 ± 1.41) scored the lowest.

In the individual item‐level analysis, the three highest‐performing behaviors all belonged to the alarm response dimension: “upon receiving an alarm signal, I respond immediately” (4.46 ± 0.88), “when multiple alarms occur simultaneously, I prioritize responses based on alarm levels” (4.38 ± 0.97), and “I respond to all alarms in my assigned area” (4.34 ± 0.97). In contrast, the three lowest‐performing behaviors were “if I believe an alarm is false or a nuisance alarm, I do not respond” (2.77 ± 1.44), “when false or nuisance alarms occur frequently, I mute or turn off alarms” (2.79 ± 1.44), and “I have missed alarms due to not hearing the alarm sound” (2.81 ± 1.40).

### 3.3. Correlation Analysis of Alarm Knowledge, Attitude, Alarm Fatigue, Burnout, Resilience, and Nurses’ Clinical Alarm–Related Behaviors

Table 3 presents the Pearson correlation coefficients between nurses’ clinical alarm–related behaviors and several related psychological and professional constructs, including knowledge, attitude, alarm fatigue, burnout, and resilience. Nurses’ alarm‐related behavior showed a strong positive correlation with alarm fatigue (*r* = 0.9607, *p* < 0.001) and a moderately strong positive association with burnout (*r* = 0.5909, *p* < 0.001). In contrast, behavior was negatively correlated with clinical alarm–related knowledge (*r* = −0.2478, *p* < 0.001). Alarm fatigue and burnout were also strongly positively interrelated (*r* = 0.6095, *p* < 0.001). Resilience demonstrated small‐to‐moderate positive correlations with all other variables (ranging from r = 0.1325 to 0.3468, all *p* < 0.01 or < 0.001).

**TABLE 3:** Correlation analysis between nurses’ clinical alarm–related behaviors with clinical alarm–related knowledge, attitude, alarm fatigue, burnout, and resilience.

	Mean ± SD	Behavior	Knowledge	Attitude	Alarm fatigue	Burnout	Resilience
Behavior	68.89 ± 9.72	1					
Knowledge	10.30 ± 1.56	−0.2478^∗∗^	1				
Attitude	49.62 ± 5.87	0.0467	0.1907^∗∗^	1			
Alarm fatigue	81.86 ± 18.95	0.9607^∗∗^	−0.2141^∗∗^	−0.0137	1		
Burnout	69.85 ± 13.14	0.5909^∗∗^	−0.1726^∗∗^	−0.0660	0.6095^∗∗^	1	
Resilience	30.91 ± 7.72	0.2265^∗∗^	0.1325^∗^	0.2638^∗∗^	0.1716^∗∗^	0.3468^∗∗^	1

^∗^ *p* *< 0.01.*

^∗∗^ *p* *< 0.001.*

### 3.4. Multiple Linear Regression Analysis of Factors Associated With Clinical Alarm–Related Behaviors

Table 4 shows the multivariate linear regression analysis. The model explained a substantial proportion of variance in alarm‐related behaviors (*R* ^2^ = 0.673; adjusted *R* ^2^ = 0.662). The analysis identified several independent factors related to alarm‐related behaviors. Working in the ICU remained a strong positive factor (*β* = 5.47, 95%CI: 0.61 to 10.33, *p* = 0.027). Implemented alarm management protocol (*β* = 4.54, 95%CI: 2.83 to 6.24, *p* < 0.001) and experienced adverse events in last years (*β* = 2.02, 95%CI: 0.28 to 3.75, *p* = 0.023) were also strong positive factors.

**TABLE 4:** Multiple linear regression analysis of factors associated with clinical alarm–related behaviors.

Variable	*B*	SE	95% CI lower bound	95% CI higher bound	*p* value
Age	−0.02	0.13	−0.27	0.23	0.855
Gender					
Male	Ref				
Female	1.35	0.87	−0.36	3.06	0.121
Professional title					
Enrolled nurse	3.95	2.97	−1.89	9.80	0.184
Junior nurse	2.97	2.97	−2.87	8.82	0.318
Charge nurse	Ref				
Senior nurse	4.44	3.24	−1.93	10.80	0.171
Department					
ICU	5.47	2.47	0.61	10.33	0.027
PICU	Ref				
NICU	2.46	2.70	−2.84	7.76	0.362
PACU	1.67	2.49	−3.23	6.57	0.504
Years of nursing experience	−0.39	0.18	−0.75	−0.03	0.032
Years in critical care unit	0.46	0.25	−0.02	0.94	0.063
Patients/shift	0.08	0.07	−0.06	0.21	0.259
Implemented alarm management protocols					
Yes	4.54	0.87	2.83	6.24	< 0.001
No	Ref				
Experienced adverse events in last years related to clinical alarms					
Yes	2.02	0.88	0.28	3.75	0.023
No	Ref				
Resilience score	0.18	0.06	0.07	0.29	0.001
Knowledge score	−0.93	0.28	−1.47	−0.38	< 0.001
Attitude score	0.36	0.07	0.21	0.51	< 0.001

Among psychological factors, resilience (*β* = 0.18, 95% CI: 0.07 to 0.29, *p* = 0.001) and positive attitude (*β* = 0.36, 95%CI: 0.21 to 0.51, *p* = 0.001) were associated with improved behaviors. However, two inverses’ associations were also identified: each additional year of nursing experience was associated with a decline in behavioral scores (*β* = −0.39, 95% CI: −0.75 to −0.03, *p* = 0.032), and higher knowledge scores were independently associated with lower behavioral score (*β* = −0.93, 95% CI: −1.47 to −0.38, *p* < 0.001).

## 4. Discussion

This research evaluated nurses’ clinical alarm–related behaviors in Malawi and identified their associated factors. The mean behaviors score (68.89 ± 9.72) indicates a moderate level of performance with room for improvement. This score is higher than a comparable Chinese study (65.14 ± 7.95) [18]. Varying and inconsistent alarm‐related behaviors have also been documented among Palestine and Korean ICU nurses [3, 5, 24]. Collectively, these observations suggest that clinical alarm management remains a widespread challenge in critical care units, with performance often falling in the moderate range. Malawian nurses’ scores are broadly comparable to those documented in better‐resourced settings, reinforcing the universality of this problem.

Findings showed the strong positive correlation with alarm‐related behaviors and both alarm fatigue and burnout, a pattern partially supported by previous studies [2, 25]. This association necessitates a paradigm shift in interventions, moving beyond individual training to addressing systemic burnout and the alarm‐laden acoustic environment [26]. While this association aligns with the intense alarm burden characteristic of resource‐constrained Malawian critical care units, it raises important methodological considerations. Both scales rely on nurses’ perceptions of their own practices and experiences, which may inflate correlations due to common response biases or overlapping item content. The exceptionally high correlation between alarm fatigue and better alarm‐related behavior is, therefore, most likely an artefact of shared method variance and item‐content overlap and should be interpreted with caution. Future studies should incorporate objective behavioral observation or ecological momentary assessment to disentangle the two constructs and better inform targeted interventions.

The departmental variation observed, where ICU nurses demonstrated higher clinical alarm–related behavioral scores than those in other units, aligns with previous research [18]. This may be attributed to the intensified clinical necessity of monitoring in adult ICUs, where higher patient acuity and greater technological density mandate more rigorous alarm recognition and management [26, 27].

Contrary to expectations, greater nursing experience was negatively associated with clinical alarm–related behavioral scores. Rather than reflecting reduced competence, this finding may indicate desensitization to alarms following prolonged exposure, a mechanism previously described as a core driver of alarm fatigue or the persistence of habitual practices acquired in less technology‐intensive care settings [4]. Similar inverse associations between experience and alarm‐related vigilance have been reported elsewhere, suggesting that experience alone does not translate into optimal alarm management practices without reinforcement through updated training [28]. These results underscore the importance of specialized, experience‐tailored refresher training focused on contemporary alarm management practices. Future mixed‐methods research should explore the underlying mechanisms in resource‐limited critical care environments.

The strong positive association between availability of implemented alarm management protocols and improved nurses’ clinical alarm–related behaviors underscores the critical importance of structured guidelines, consistent with previous findings [1]. Such protocols provide clear directives, enhance nurses’ confidence, and contribute to a more organized approach to alarm response, thus improving overall behavioral scores. Training programs grounded in behavioral frameworks such as the theory of planned behavior have been shown to reduce nonactionable alarms and lower alarm fatigue among critical care nurses, further supporting the behavioral impact of structured approaches [29–31].

Nurses who had experienced alarm‐related adverse events in the past 2 years demonstrated higher alarm related behavioral scores, suggesting a potential learning effect. This finding is consistent with the concept of incident‐informed vigilance, whereby direct experience of patient harm due to missed or delayed alarms may heighten awareness of alarm criticality and motivate more diligent alarm management practices [32]. Importantly, this association suggests that structured, nonpunitive postincident debriefing frameworks make this learning process explicit and systematic rather than incidental [1].

Resilience was positively associated with nurses’ clinical alarm–related behaviors. Nurses with higher resilience may be better equipped to sustain attentive, proactive alarm management despite the cognitive overload and emotional exhaustion that characterize high‐alarm‐burden environments [24]. This is supported by previous research which demonstrates that resilience mediates the relationship between alarm fatigue and burnout among critical care nurses [33]. These findings suggest that strategies fostering resilience, such as structured peer support, mentorship, and mindfulness‐based stress reduction integrated into existing in‐service education, may be valuable complements to protocol‐based interventions.

Knowledge–practice gaps are well documented in critical care nursing, where contextual barriers such as high workload, frequent false alarms, limited staffing, and weak institutional support often prevent nurses from translating theoretical knowledge into practice [2, 33, 34]. These contribute to alarm fatigue and suboptimal alarm‐related behaviors. Alternatively, this inverse association may partly reflect measurement overlap between the knowledge and behavior scales, or reverse causation, whereby nurses with poorer behaviors seek out more knowledge to compensate. Future mixed‐methods research should examine how knowledge interacts with contextual constraints to shape alarm‐related behaviors in resource‐limited environments.

A positive attitude score was independently associated with better clinical alarm–related behaviors. This suggests that a favorable disposition toward alarm management and its importance for patient safety is crucial for driving positive actions [35]. Nurses who perceive clinical alarms as important and who are motivated to manage them effectively are more likely to exhibit desirable clinical alarm–related behaviors [1, 35, 36]. These findings highlights the importance of fostering a positive attitude through education that emphasizes the value of alarms and their role in patient outcomes, rather than just providing factual knowledge [1].

### 4.1. Strengths and Limitations

This study offers several strengths in understanding nurses’ clinical alarm–related behaviors, including the identification of specific factors such as department, experience, protocols, and past adverse events, which were analyzed using robust statistical methods (multivariate linear regression). The detailed examination of these factors, together with resilience, knowledge, and attitude, provides a comprehensive view of the influences on alarm management.

However, the study also has limitations. The cross‐sectional design precludes the establishment of causal relationships; this study cannot definitively conclude, for example, that a more positive attitude causes better alarm behaviors, only that the two are associated. The self‐reported nature of the behavioral data means findings reflect nurses’ perceptions of their own practices, which may diverge from objectively measured behavioral performance, which introduces the potential for common‐method bias. Future studies using observational or electronic monitoring methods would complement these self‐report findings. In addition, the inclusion of only one private institution alongside four public tertiary hospitals may limit generalizability to other types of facilities.

### 4.2. Implications for Nursing Management

The findings of this study offer several specific, actionable implications for nursing management in resource‐limited critical care settings. Interventions can be categorized into individual‐level and system‐level strategies.

At the individual level, nurse managers should implement targeted refresher training programs specifically for experienced nurses (> 5 years), focusing on alarm recognition, prioritization, troubleshooting, and appropriate responses to false/nuisance alarms. These programs should be nonpunitive and framed around evolving technology. Managers should also promote positive attitudes toward alarms and build resilience through peer recognition programs and brief monthly peer‐support or mindfulness sessions integrated into existing in‐service schedules.

At the system level, managers and hospital administrators should prioritize the development, implementation, and regular review of standardized alarm management protocols. These protocols should clearly define alarm parameter customization, response prioritization, escalation pathways, and nuisance alarm handling. Implementation should be supported by quarterly alarm audits using available monitoring system logs and structured, nonpunitive debriefing sessions within 48 h of any alarm‐related adverse event.

These recommendations are designed to be feasible in resource‐constrained environments such as Malawi. Protocol development can draw upon existing WHO and Ministry of Health templates at minimal cost, while training and resilience activities can be integrated into current in‐service education without additional resources. Collectively, these strategies can enhance nurses’ alarm‐related behaviors and contribute to improved patient safety.

## 5. Conclusion

Therefore, findings of this study suggest the need for multifaceted approach that considers not only the implementation of protocols but also the psychological and experiential aspects associated with nurses’ clinical alarm behaviors. While experience can sometimes lead to desensitization, adverse events and resilience may enhance vigilance. Crucially, the regression analysis indicated that a positive attitude was associated with more appropriate clinical alarm–related behaviors, potentially to a great extent than knowledge alone. Future interventions should focus on comprehensive strategies that integrate protocol adherence, psychological support, and a positive attitudinal shift to optimize nurses’ responses to clinical alarms and enhance patient safety.

**Nomenclature**
SSA: Sub‐Saharan Africa
SEIPS: Systems engineering initiative for patient safety
ICU: Intensive care unit
NICU: Neonatal intensive care unit
PICU: Pediatric intensive care unit
PACU: Postanesthesia care unit
SD: Standard deviations
MBI: Maslach Burnout Inventory
CI: Confidence interval
WHO: World Health Organization

## Author Contributions

Chimwemwe Joyce Kamuyalo, Bingyu Li, and Liqing Yue conceptualized the idea and prepared the protocol for data collection. Chimwemwe Joyce Kamuyalo, Mtoro J. Mtoro, and Elihuruma Eliufoo Stephano collected data and conducted the formal analysis. Chimwemwe Joyce Kamuyalo, Liqing Yue, Bingyu Li, Elihuruma Eliufoo Stephano, and Mtoro J. Mtoro operationalized the idea, supported interpretation, prepared the first draft of the manuscript, and reviewed subsequent versions of the manuscript.

## Funding

This work was supported by General Program of the National Natural Science Foundation of China (72174209 and 72174316).

## Disclosure

All authors read and approved the final manuscript.

## Ethics Statement

This study protocol was reviewed and approved by Institutional Review Board of Central South University, Xiangya School of Nursing and awarded approval number 2025WJ021. The protocol was locally reviewed and approved by National Health Science Research Center in Malawi (protocol number 25/09/4740). Before the official commencement of the study, institutional approval from all 5 participating hospitals was secured. Support letters were collected from each participating hospital to authorize the implementation of the research in their facilities. All eligible participants were informed about the purpose, procedures, risks, and benefits of the study. Participants were asked to voluntarily provide consent prior to participation. The principal investigator together with trained research assistants facilitated the consent process and addressed any questions raised by the participants.

## Consent

The authors have nothing to report.

## Conflicts of Interest

The authors declare no conflicts of interest.

## Supporting Information

Additional supporting information can be found online in the Supporting Information section.

## Supporting information


**Supporting Information** STROBE Statement—Checklist of items that should be included in reports of cross‐sectional studies

## Data Availability

The raw data supporting the conclusions of this article will be made available by the authors without undue reservation via contact to the first author’s email (kamuyaloc@gmail.com).
